# The Use of Combining Ability Analysis to Identify Elite Parents for *Artemisia annua* F1 Hybrid Production

**DOI:** 10.1371/journal.pone.0061989

**Published:** 2013-04-23

**Authors:** Theresa Townsend, Vincent Segura, Godfree Chigeza, Teresa Penfield, Anne Rae, David Harvey, Dianna Bowles, Ian A. Graham

**Affiliations:** 1 Centre for Novel Agricultural Products, Department of Biology, University of York, York, United Kingdom; 2 Institut National de la Recherche Agronomique, UR0588, Orléans, France; 3 Agricultural Research Council: Grain Crops Institute, Potchefstroom, South Africa; 4 Research and Knowledge Transfer, University of Exeter, Exeter, United Kingdom; 5 Genetics Department, Cherry Valley Farms Ltd., Caistor, United Kingdom; University of Umeå, Sweden

## Abstract

*Artemisia annua* is an important medicinal crop used for the production of the anti-malarial compound artemisinin. In order to assist in the production of affordable high quality artemisinin we have carried out an *A. annua* breeding programme aimed at improving artemisinin concentration and biomass. Here we report on a combining ability analysis of a diallel cross to identify robust parental lines for hybrid breeding. The parental lines were selected based on a range of phenotypic traits to encourage heterosis. The general combining ability (GCA) values for the diallel parental lines correlated to the positive alleles of quantitative trait loci (QTL) in the same parents indicating the presence of beneficial alleles that contribute to parental performance. Hybrids generated from crossing specific parental lines with good GCA were identified as having an increase in both artemisinin concentration and biomass when grown either in glasshouse or experimental field trials and compared to controls. This study demonstrates that combining ability as determined by a diallel cross can be used to identify elite parents for the production of improved *A. annua* hybrids. Furthermore, the selection of material for breeding using this approach was found to be consistent with our QTL-based molecular breeding approach.

## Introduction

Malaria is a global health problem with more than 1 billion people living in high risk areas. In response to the increasing levels of resistance to many monotherapy treatments the World Health Organisation recommends that artemisinin-based combination therapies (ACTs) should be used as the first-line treatment of uncomplicated *Plasmodium falciparum* malaria [1] [2]. Artemisinin is a sesquiterpenoid synthesized in the glandular trichomes of *Artemisia annua* (asteraceae). *A. annua* is native to Asia where it is cultivated as a crop in China and Vietnam but has been introduced across a wide range of countries and is also cultivated in Africa and India. The plant is a short day annual which is naturally cross pollinated. Its use as a herbal remedy for the treatment of malaria, and its effects have been well documented in traditional Chinese herbal medicine for over 2000 years [3] [4] [5]. Although semi-synthetic synthesis of artemisinin has recently moved into commercial production and distribution [6], it is expected that agricultural production will remain a crucial source of artemisinin for ACTs for the foreseeable future. However the yield of artemisinin produced by *A. annua* is low, and with increased demand for ACTs, there are concerns that the existing supply chain will be unable to produce consistent, affordable levels of the chemical in the quantities required. One way to increase artemisinin availability is to develop improved varieties of *A. annua*. These improvements would benefit the existing supply chain by improving confidence in the crop, stabilizing supplies and reducing costs [7].

*A. annua* is relatively undeveloped as a crop although it has been documented that the artemisinin content in the dry leaf of varieties from different geographical origins varies considerably, ranging from <0.01 to >1.0% [8] [5] [9]. This variation can be attributed to an extent to differences in farming practices, periods of harvest and also environmental factors such as temperature and nutrient availability [5] [10] [11]. However, artemisinin content has also been shown to be highly heritable, indicating that a strong genetic component contributes to the variation seen in the cultivated crop [10] [9]. Such genetic components and their interactions with non-genetic components can be exploited for breeding purposes to produce improved hybrid lines.

We recently reported the first genetic linkage map for *A. annua* and described Quantitative Trait Loci (QTL) that account for a significant amount of the variation in a number of traits that impact on artemisinin yield, which we calculated as a product of artemisinin concentration and plant fresh weight [12]. Prior to the tools being available for molecular breeding, hybrid development based on traditional breeding methods had been carried out by a number of organisations including Mediplant in Switzerland, The University of Campinas, Brazil and the Central Institute of Medicinal and Aromatic Plants, India. Mediplant have been breeding *A. annua* varieties since 1989 and are the producers of one of the current market leaders, ‘Artemis’ an F1 hybrid [13] [5]. The University of Campinas, in collaboration with Mediplant, have developed hybrids specifically for growth in Brazil and reported increases of artemisinin from 5 kg/ha to approximately 25 kg/ha [14]. The Central Institute of Medicinal and Aromatic Plants have also developed their own hybrids, including CIM-Arogya, with a reported artemisinin concentration of 1.2% [15].

In parallel with establishing the QTL map and molecular marker platform for the improvement of *A. annua* we also screened for artemisinin content in 23,000 individuals from an F2 population that carried a considerable amount of genetic variation [12]. To evaluate the suitability of the resulting material from this screen as parents for hybrid seed production a diallel cross programme was performed on a subset of lines that were selected entirely on phenotypic variation. The diallel method is commonly used by plant geneticists to gain information on genetic parameters and so aid parent selection for hybrid production [16] [17] [18] [19]. The approach we have taken is to analyse the diallel cross based on the methods of Griffing [16] which have been widely used to estimate combining ability to identify superior parents for hybridization. The analysis provides information on the main effects (general combining ability; GCA) and interactions (specific combining ability; SCA) between parental lines [20] [21] [22]. This method has been used extensively in the breeding of many economic crops including for example corn, wheat and cotton [23] [24] [25] [26] [27]. Combining ability analysis in A. *annua* has also been previously carried out by Delabays on *A. annua* clones [28] [9] based on Griffing’s methods. This analysis also showed broad sense heritability to be high; indicating the trait was governed by genetic factors and that narrow sense heritability was also relatively high [28] [9]. This suggested that parents selected for their high artemisinin content would generate improved progeny.

Here we present results from our diallel cross on *A. annua* which was carried out to identify parental lines with good combining ability that could be used in future crossing strategies. A subset of 48 hybrids from the diallel was selected based on performance under glass and these have now been tested in field trials to study genotype by environment interactions. Comparison of outputs from the diallel-based and molecular marker-based approaches revealed that common parental lines have been selected from the two approaches.

## Materials and Methods

### Forward Screen Population

**Figure 1:**
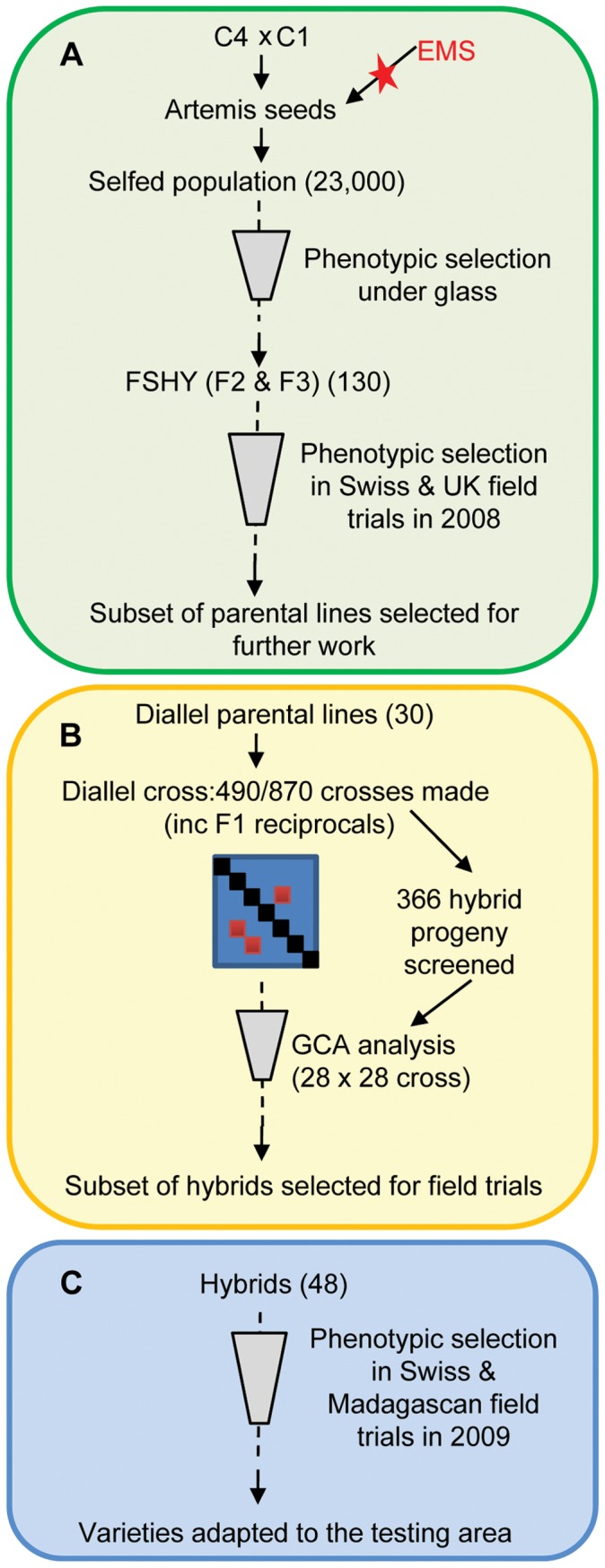
Overview of work schedule followed to evaluate parental lines and hybrid progeny. Each box illustrates steps taken in selecting parental lines and their hybrid progeny for further work. A) Forward screen: the evaluation of Forward Screen High Yielding (FSHY) individuals and selection of parental lines for the diallel cross based on phenotypic variation. B) Diallel cross: summary of the diallel cross combining ability analysis on the 366 hybrids screened and estimated general combining ability (GCA) values for the 30 diallel parental lines, enabling selection of parental lines for future crossing strategies. C) Experimental field trials: testing of promising hybrid progeny from the diallel at two field locations and selection of hybrids from this for further field trials in a greater range of growing areas.

The diallel cross was set up with 30 *A. annua* parental lines selected from F2 material that had been derived from an ethyl methane sulphonate mutagenised population of F1 Artemis seed as previously described [12] (Figure 1A). Metabolite profiling for artemisinin concentration in F2 plants grown under glass was used to identify the top 1% of individuals from this population, herein described as ‘forward screen high yielding’ or ‘FSHY’. These FSHY individuals have been maintained through vegetative propagation at the University of York. This material can be accessed for research purposes by contacting the corresponding author.

The selection of parental lines for the diallel was from a subset of 130 of the FSHY lines based on three phenotypic characteristics (flowering time, leaf area and trichome density) with the aim of maximising phenotypic diversity within the parental genotypes. Data were recorded from material grown under glass for 12.5 weeks and are shown in Table S1. Genomic DNA from the FSHY individuals was included on the Illumina genotyping platform alongside the F1 mapping population as previously described [12]. Table S2 shows the heterozygosity values calculated for the diallel parental lines based on the SNP segregation data presented in [12]. The markers associated with yield and leaf area QTL used for calculating QTL scores are also presented in Table S3.

The 30 lines selected for the diallel had previously been evaluated for artemisinin levels and biomass as part of a larger group of 130 genotypes tested in FSHY field trials carried out in Conthey, Switzerland (46°13′N latitude, 7°17′E longitude and 485 m above sea level) and York, UK (53°5′N latitude, 1°7′W longitude and 5 m above sea level) in April 2008 [12].

### The Diallel Cross

The diallel cross was set up in August 2008 in glasshouse facilities at Stockbridge Technology Centre Ltd York, UK (Figure 1B). Rooted cuttings of the parental lines were grown in four inch pots in 16 hours of daylight for 12 weeks prior to being moved into 12 hours of daylight to induce flowering as previously described [12]. From 870 possible cross combinations (including reciprocals but not parental inbreds) a single cross of 490 was achieved; 366 of these yielded enough seed to be grown up for screening (156 of these were reciprocals). The crosses and seed harvesting were carried out following the methods previously described [12]. Seed cleaning was carried out by separating the seeds from the capitulum using 850 and 600 µm sieves. Seeds were then collected, labelled and stored at 10°C.

### Hybrid Screening Under Glass

The 366 crosses were divided into three batches and seeds were sown out in December 2008, February 2009 and March 2009 in glasshouse facilities at the University of York. Artemis seeds were sown as a control to assess possible environmental effects within each batch. Twenty seedlings from each of the 366 crosses were pricked out into p40 trays in Levington F2– seed and modular compost, and grown under long day conditions (16 hrs) at 22°C max 17°C min. After 12 weeks leaf samples were collected from the plants for metabolite analysis and all plants were scored for height in centimetres and branch number. Leaf samples were collected from a subset of the plants (five of the 20 plants from each cross) for leaf area (mm^2^) and trichome density measurements. The methods used for metabolite analyses, leaf area and trichome density measurements are as previously described [12]. The data collected from the screening was then used for carrying out combining ability analysis as described below.

### Hybrid Field Trials

A subset of 48 hybrid lines resulting from the diallel cross programme were selected on the basis of their performance under glass and seed availability and sent to Mediplant, a Swiss research centre for aromatic and medicinal plants based in Conthey Switzerland (46°13′N latitude, 7°17′E longitude and 485 m above sea level) and Bionexx, a commercial *A.annua* grower with field sites in several locations in Madagascar including Antsahamaroloha, (19° 51′S latitude, 47° 09′E longitude and 1653 m above sea level). Experimental field trials were conducted in 2009 by both Mediplant and Bionexx (Figure 1C). Climatic data for these sites is provided in Table S8.

### Switzerland Trial

In Switzerland, two replicates of the 48 hybrid lines alongside controls (Artemis and a local check specific to Mediplant) were sown in the glasshouse in April 2009. When plants were seven weeks old 20 plants per plot were transplanted out into the field following a randomized complete block design. The plants were grown in the field for six months before being manually harvested. Prior to harvest, plant height was measured and harvesting was then carried out by cutting all plants at ground level. The fresh weight for each plant was recorded before the plant material was oven dried (30°C for two weeks until material reached a level of <10% moisture). After drying, the leaves were stripped and a dried leaf weight per plot recorded. For each plot, the dried leaf material was pooled and mixed thoroughly after which four random samples totalling 100 g were collected from the pooled material and sent to York for artemisinin and metabolite quantification. The metabolite profiles were generated from four technical replicate samples of 10 mg of ground dried tissue sampled from the 100 g of pooled material. The powdered samples were extracted in 96 well plate format by gently shaking in a final volume of 500 µl comprising 480 µl 9∶1 chloroform/ethanol and 20 µl internal standards for 1 hour. Following extraction the samples were spun for 1 min at 4350 rpm and allowed to stand for 10 min before the top layer was removed and diluted with four parts ethanol. Following their preparation, extracts were analysed according to the method previously described [12].

### Madagascar Trial

In Madagascar, three replicates of the same 48 hybrid lines alongside controls (Artemis, included in the trial with permission from Mediplant, Switzerland and a local variety commercially grown in Madagascar) were sown into trays containing soil outside in April 2009. After six to seven weeks 32 plants per plot were transplanted into the field following a randomized complete block design. The plants were then grown in the field for six months before plants from the net plot only (12 plants from the centre of each plot) were manually harvested. Plant height was measured prior to harvest and harvesting was then carried out by cutting plants at ground level, after which the fresh weight for each plant was recorded before the plant material was allowed to dry in the field. After drying, the leaves were stripped and a dried leaf weight per plot recorded. For each plot, the dried leaf material was pooled and mixed thoroughly after which four random samples totalling 100 g were collected from the pooled material and sent to York for artemisinin and metabolite quantification. The metabolite profiles were generated from four technical replicate samples of 10 mg of ground dried tissue sampled from the 100 g of pooled material according to the extraction and analysis method described above.

From both the Swiss and Madagascan data sets the plant leaf yield was calculated from the dry leaf weight/plot measurements. This was in turn used to calculate the projected dry leaf weight expressed as kg/ha based on the planting density. This value was then used to calculate the projected yield of artemisinin in kg/ha (artemisinin concentration × dry leaf weight). These values were then incorporated into the analysis described below.

### Data Analysis

All data analysis was carried out using the SAS v9.2 software (SAS Institute Inc, Cary, North Carolina, USA). Prior to the analysis the distribution of the trait data collected was first assessed using the PROC UNIVARIATE procedure. Traits deviating from the normal distribution according to the Shapiro-Wilk test were transformed with either a natural logarithm or square root to reach a normal distribution.

### FSHY Genotype Field Data

Data from FSHY field trials conducted in 2008 [12] were analysed to estimate performance of 130 FSHY individuals from which 30 parental lines had already been chosen, based on phenotypic characteristics alone, for the diallel cross programme. Data analysis was performed using the following mixed linear model based on combining the data from both Swiss and UK 2008 field trials [12] and including the effect of site into the model and its interaction with the genotype effect:
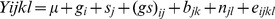
(1)Where Y*_ljk_*, is the observed performance of the repetition *l* of the *i*th genotype in the block *k* of site *j*, µ is the overall mean, g*_i_* is the random effect of genotype *i*, s*_j_* is the fixed effect of the site *j*, (gs)*_ij_* is the random interaction between the genotype *i* and the site *j*, b*_jk_* is the fixed effect of the block *k* nested into site *j*, n*_jl_* is the fixed effect of the neighbour of the plant *l* nested in the site *j* and e*_ijkl_* is the random residual term. The neighbour co-variable n*_jl_* was calculated as proposed by Papadakis [29], *i.e.* the mean of the residuals from the following model: Y*_ijl_* = µ+g*_i_*+e*_il_* calculated within each site on the neighbours of the repetition *l* of genotype *i*, according to grids of 3×3, 3×5, 5×3 and 5×5 meters around the repetition *l*. For most of the studied traits, the block and neighbour effects appeared to be redundant. Thus, in order to get the best model, for each trait a model selection procedure based on the Bayes Schwarz information criterion (BIC) was carried out in model 1 including the block effect and the neighbour co-variable and removing iteratively the least significant effect until the BIC reaches a minimum. In the selected models, restricted maximum likelihood (REML) estimates of genotype and genotype by site interaction and residual variance components were computed, and best linear unbiased predictors (BLUPs) of the hybrid and hybrid by site interaction effect were extracted to be used as descriptors of the genotype performances within and between locations respectively.

### Combining Ability Analysis of the Diallel Cross

To estimate the variance components of the diallel cross from plants grown under glass the following linear model was fitted to the data based on Griffing’s method 3which includes the progeny of the crosses and their F1 reciprocals but not the parents [16].
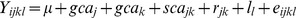
(2)Where, Y*_ijkl_*, is the *i*-th observation of the *l*-th batch for the *jk*-th cross, µ, is the overall mean, gca*_j_* and gca*_k_* are the GCA effect of the *j*-th female and the *k*-th male parent respectively, sca*_jk_* is the SCA effect for the *jk*-th cross, r*_jk_* is the reciprocal effect for the *jk*-th cross, l*_l_* is the effect of the *l*-th batch and e*_ijkl_* is the random error term. The GCA, SCA, reciprocal effects, and error term were considered random to allow an estimation of their variance whilst the effect of batch was considered fixed to account for some potential bias induced by carrying out the experiment in batches. From the above model, variance estimates were computed for each random term. To evaluate the GCA values of parental lines, BLUPs of the GCA effects were also computed. The computation was performed using REML in the PROC MIXED procedure [30] [18] [19].

### Field Trial Hybrid Evaluation

Hybrid data from 2009 Swiss and Madagascan field trials were analysed to estimate performance of the selected hybrids both within each single field trial and combining the data from both field trials, using the mixed linear models 3 & 4 respectively;
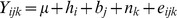
(3)Where Y*_ijk_*, is the observed performance of the repetition *k* of the *i*th hybrid in the block *j*, µ is the overall mean, h*_i_* is the random effect of hybrid *i*, b*_j_* is the fixed effect of the block *j*, n*_k_* is the fixed effect of the neighbour of plant *k* (computed using the Papadakis method as described for Model 1) and e*_ijk_* is the random residual term. As previously described, a model selection procedure based on the BIC was carried out for each studied trait including either, the block effect and/or the neighbour co-variable within each site. In the selected models REML estimates of hybrid and residual variance components were computed and BLUPs of the genotypic effect were extracted and used to identify hybrid lines performing well at each location. Model selection and estimation were done using the mixed procedure of SAS v.9.2 software.

The second model for hybrid data analysis (Model 4) is based on combining both data sets and including the effect of site into the model and its interaction with the hybrid effect:
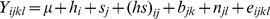
(4)Where Y*_ijkl_*, is the observed performance of the repetition *l* of the *i*th hybrid in the block *k* of site *j*, µ is the overall mean, h*_i_* is the random effect of hybrid *i*, s*_j_* is the fixed effect of the site *j*, (hs)*_ij_* is the random interaction between the hybrid *i* and the site *j*, b*_jk_* is the fixed effect of the block *k* nested into site *j*, n*_jl_* is the fixed effect of the neighbour of plant *l* nested in site *j* (computed within each site using the Papadakis method as described above) and e*_ijkl_* is the random residual term. As described above, a model selection procedure based on the BIC was carried out including either, the block effect and/or the neighbour co-variable within each site. In the selected models, REML estimates of hybrid, hybrid by site interaction and residual variance components were computed, and BLUPs of the hybrid and hybrid by site interaction effect were extracted to be used as descriptors of the hybrid performances between and within locations. It should be noted that for models 3 and 4 the effect of h may not be completely independent due to some hybrids sharing parents.

## Results

### Evaluation of FSHY Individuals

Genotype (G) and Genotype by Environment (G×E) analysis was carried out on the data collected from field trials conducted in 2008 at York, UK and Conthey, Switzerland with 130 FSHY individuals, including the 30 parental lines that were selected for the diallel cross. The analysis showed that, despite having been selected on the basis of high artemisinin concentration under glass, a significant genotype effect (p<0.01) was also observed for artemisinin concentration in the field. In addition significant genotype effects (p<0.01) were found for the other recorded traits, with the largest effects being for fresh weight and plant height (Table 1). Significant (p<0.01) GxE interactions were also noted for height, branch number and fresh weight. The GxE effect however explained less than 20% of the phenotypic variance, the exception being artemisinin concentration where both G and GxE effects explained a similar proportion of phenotypic variance (Table 1).

**Table 1:** Restricted maximum likelihood (REML) analysis variances calculated from G×E model showing the significant genotype (G) and genotype × environment (G×E) interactions found when comparing 130 FSHY individuals trialled in the field in Switzerland and UK in 2008.

Source	Artemisinin concentration (µg/mg)	Fresh weight (g)	Height (cm)	Number of branches
G	1.80**** (27)	321667.00**** (48)	172.27**** (44)	31.02**** (36)
GxE	1.58**** (24)	102417.00**** (15)	67.03**** (17)	10.65**** (12)
Error	3.27 (49)	239801.00 (36)	152.87 (39)	45.02 (52)

**** indicates significance at <0.001 level; values in parentheses represent percent variance.

### Analysis of Hybrids Derived from the Diallel Cross

**Figure 2:**
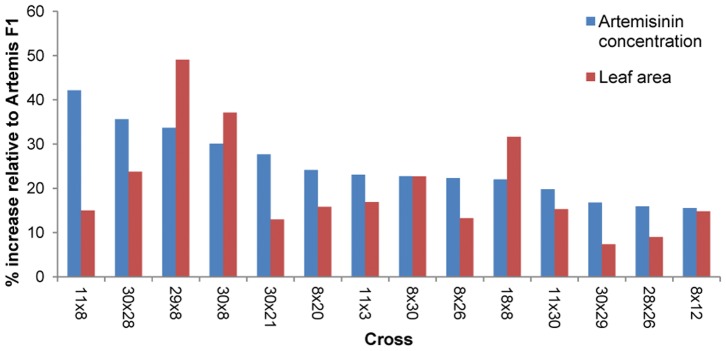
Artemisinin concentration (blue) and leaf area (red) for a subset of diallel hybrids. Values shown represent the percentage increase in artemisinin concentration and leaf area, over Artemis F1, for the 14 best performing hybrids produced from the diallel cross programme when tested under glass. The parental lines which were crossed to produce each hybrid are shown. Values were calculated from a minimum of five individual replicates.

From the 30 parent lines selected for the diallel cross 28 were successfully crossed, with two parental lines, parents 23 and 25, being removed due to lack of synchrony in flowering time with other lines. From the 28×28 diallel a total of 366 hybrids yielded enough seed to perform a screen of hybrid material under glass. From the range of artemisinin concentrations and leaf area seen under glass it was clear that some hybrids performed above the levels of the Artemis control and others below (Table S4). Figure 2 shows the performance of a subset of hybrids that performed better in comparison to Artemis with 10–40% increases in artemisinin concentration and between 5 and 45% increases in leaf area.

Combining ability analysis based on Griffing’s method 3 was carried out on 342 of the hybrids derived from the diallel crosses and the variances calculated from this are presented in Table 2. The analysis showed significant effects for GCA values (p<0.01) for all traits while the effects for SCA were not significant. Interestingly, the reciprocal effects were found to be significant (p<0.001) for all traits raising the possibility of maternal effects.

**Table 2:** Combining ability variances calculated for a 28×28 diallel for *Artemisia annua* hybrids grown under glass.

Source	Artemisinin concentration (µg/mg)	Height (cm)	Branch number	Internode number	Leaf area (mm^2^)	Trichome density (cm^2^)
GCA	0.58****	15.39****	1.59****	0.01****	28139****	2.04****
SCA	0.30	14.07	–	0.01	–	0.87
Reciprocal	2.19****	76.86****	4.75****	0.05****	68008****	4.30****
Error	13.47	239.59	25.12	0.14	148547	24.95

**** indicates significance at <0.001 level.

BLUP values calculated from the combining ability analysis provided an estimate for GCA values for the individual diallel parent lines (Table 3).

**Table 3:** Estimates of GCA for the parent lines used in a 28×28 diallel cross.

Parent	GCA artemisinin concentration	GCA height	GCA branch number	GCA internodes	GCA leaf area	GCA trichome density
1	−0.45	−8.10****	−1.59***	−0.20****	−34.14	−1.91***
2	0.72	0.96	−1.14**	0.10	−31.82	0.35
3	−0.01	5.72***	2.18****	0.10*	−54.16	0.23
4	−0.97**	3.11	−0.62	0.12**	−40.17	−1.10
5	−0.93**	1.53	1.37**	−0.02	−268.62***	−0.35
6	0.23	−0.45	0.30	−0.04	1.38	−0.40
7	0.62	1.17	1.00	0.00	76.94	0.10
8	1.25***	5.87***	0.28	0.18****	287.18****	0.35
9	0.17	−1.94	−0.98	−0.04	2.89	0.31
10	1.02***	4.53**	1.35**	0.09	6.10	0.75
11	0.41	−2.10	−1.22**	0.01	324.92****	−1.78***
12	0.15	2.57	1.81***	−0.01	62.52	0.95
13	−0.33	−0.24	0.63	−0.03	−37.89	0.47
14	0.24	−1.39	0.39	−0.04	−41.79	2.31***
15	−0.26	−1.81	−1.49	0.01	116.38	−0.73
16	−0.05	−1.57	0.04	−0.06	−17.95	−1.30
17	0.03	−0.67	−0.83	0.02	−22.14	−0.89
18	0.05	−0.81	−0.17	−0.01	14.55	−0.17
19	−0.69	−6.79***	−1.25**	−0.19***	−305.13****	−1.29
20	−1.20***	−1.44	−0.53	−0.03	−213.08***	−1.27
21	−0.42	2.84	1.86***	0.01	−48.09	1.76**
22	−0.09	−0.39	−0.07	−0.01	52.85	−0.41
24	0.29	1.04	0.56	0.01	−104.06	0.25
26	−1.01***	−3.91**	−0.08	−0.13***	−102.27	3.31****
27	−0.17	−1.24	−0.96	0.01	−132.08	−0.94
28	0.05	2.03	0.20	0.06	16.75	0.89
29	0.06	−0.84	−1.66***	0.05	216.88***	−0.48
30	1.29****	2.32	0.63	0.05	274.04****	0.98

* indicates significance at 0.05 level,

** indicates significance at 0.01 level,

*** indicates significance at 0.001and

**** indicates significance at <0.001.

From the 28 parents, three parent lines, 8, 10 and 30 were identified with high and significant GCA values for artemisinin concentration, with parent 30 having the highest GCA. Progeny from crosses involving these three parents performed as well or better than controls regardless of whether they had been the maternal or paternal parent. These three parental lines also had significant GCA values for other traits. For example parent 8 had a significant and positive GCA score for height, internode number and leaf area; parent 10 had positive significant GCA scores for height and branch number and parent 30 had positive significant GCA scores for leaf area.

**Figure 3:**
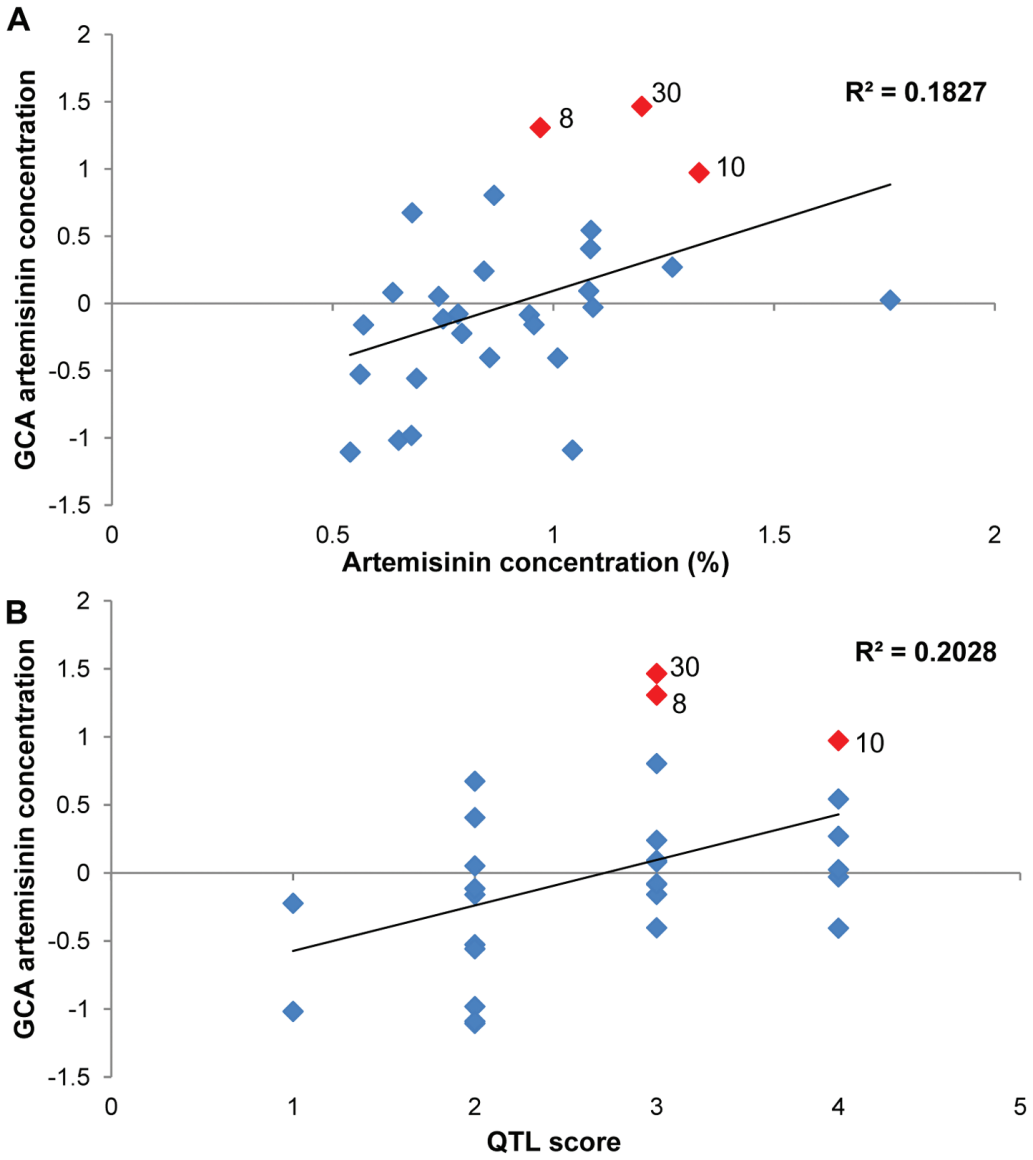
Analysis of general combining ability (GCA) values from diallel cross parental lines. A. Relationship between parental GCA values and artemisinin concentration recorded from parental lines grown in the field in the UK in 2008. B. Relationship between parental GCA values and QTL scores. Data points highlighted in red in each panel represent parent lines discussed in the text.

When comparing the UK and Swiss 2008 field data (described above) for the 30 FSHY individuals selected as parental lines for the diallel cross with the glasshouse derived data, significant correlations (p<0.05) were found between the GCA values for both artemisinin concentration and leaf area (Figure 3A). Parent 30, with the most significant GCA for artemisinin concentration in the glasshouse screen also had high artemisinin content in the field. Other traits such as leaf area and height did not show such strong correlations, highlighting the value of performing the diallel analysis to identify parents with good GCA for these other important traits (see Table 3).

QTLs on the *A. annua* genetic linkage map that associate with biomass and yield traits [12] were used to generate QTL ‘positive allele’ scores for each genotype. A Pearson’s correlation test was performed and a significant (p<0.05) correlation was found between the QTL positive allele score for yield (artemisinin content × fresh weight) and the parental GCA values for concentration (Figure 3B). A similar positive correlation between QTL scores and GCA values was observed for leaf area data (Figure S1).

### Experimental Field Trial Analysis of Hybrids

**Figure 4:**
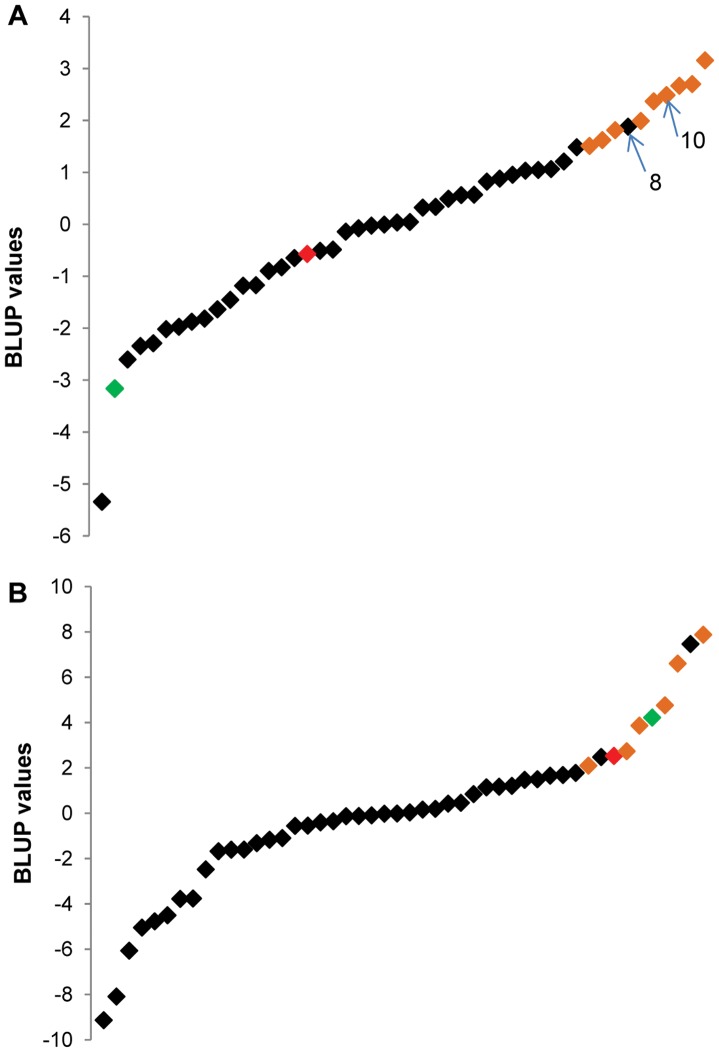
Best linear unbiased predictor (BLUP) values calculated from individual field site analyses. A. Data from the Madagascar field site. B. Data from the Switzerland field site. BLUP values are illustrate performance of 48 hybrids against local check control varieties and Artemis F1. Red markers indicate Artemis and green markers indicate the local check used at each location. Orange markers highlight hybrids within the top ten hybrids tested in each field trial which result from crosses involving parent 30. Hybrids within the top ten which resulted from a cross involving parent 8 or parent 10 are also identified.

Single site analysis of 48 hybrids grown in experimental field trials based on a randomized complete block design in Switzerland and Madagascar in 2009 revealed that the hybrids were significantly different (p<0.001) for all trait data (Table S5). From this analysis a number of hybrids were found to outperform the Artemis control variety and also the local check varieties selected for inclusion in the trial by the growers at each experimental site (Figure 4A&B). The large number of hybrids outperforming Artemis and the local check in Madagascar (Figure 4A) is particularly relevant to our breeding programme since this is a commercial production area for artemisinin.

Analysis of the parental makeup of the hybrids revealed that parent 30, which has the highest GCA score based on glasshouse derived data, was used in the production of nine hybrids found in the top ten hybrids ranked based on artemisinin yield (kg/ha) in Madagascar and six of the top ten hybrids in Switzerland (Figure 4 A&B). Furthermore, parents 8 and 10, which also had high GCA scores, had been used for the production of two of the hybrids in the top ten ranking hybrids trialled in Madagascar and these are highlighted in Figure 4A.

Combining the data from Swiss and Madagascan sites identified a significant (p<0.01) genotype effect for artemisinin concentration and plant height with the genetic variation seen in height (42%) being greater than that for artemisinin concentration (23%) (Table 4). Within the analysis the effect of site was also found to be significant with the hybrids tested at the Swiss site showing an increase in artemisinin yield and biomass (based on leaf yield) in comparison to the Madagascar site (Table S5). This combined site analysis once again demonstrates that the hybrids that performed best in the field in Switzerland and Madagascar were all derived from parents that we independently demonstrated from glasshouse data had high GCA scores. For example when looking at BLUP data derived from the combined analysis, at the Swiss site, hybrids 1086 and 1174r, which share parent 30, and hybrid 1005r, which is derived from parent 10, had significant BLUP values for artemisinin yield (Table S6). At the Madagascan site the BLUP value for artemisinin yield for hybrid 1101, which is derived from parent 30, (the parent line with the highest GCA score) was also significant.

**Table 4:** REML analysis variances calculated from a G×E model indicating significant genotype (G) and genotype × environment (G×E) interaction of hybrids field trialled in Switzerland and Madagascar in 2009.

	Plant leaf dry weight (g)	Leaf yield (Kg/ha)	Artemisinin concentration (µg/mg)	Yield (Kg/ha)	Average height (cm)
G	44.57 (11)	12144.00 (11)	0.37* (23)	1.40 (7)	97.90*** (42)
G×E	80.16* (20)	24970.00* (22)	0.57*** (35)	9.34*** (44)	64.91*** (28)
Error	278.70 (69)	76926.00 (67)	0.68 (42)	10.68 (50)	69.69 (30)

* indicates significance at 0.05 and

*** indicates significance at 0.001,values in parentheses represent the percentage variance in each case.

To establish the impact of trial site environment on hybrid performance we also evaluated GxE interactions. A significant GxE effect (p<0.05-p<0.01) was found for all traits between the sites and hybrids (Table 4). This effect was largest (∼35–45%) for artemisinin concentration and yield indicating that the trial site environment was having a large impact on these traits. For plant height the GxE effect was also reasonably large at 28%, while for plant leaf dry weight the effect was slightly lower at ∼20%. Climate and temperature differences at the two sites are just two of the possible factors responsible for the large GxE effect (Table S7).

## Discussion

### Evaluation of Parents for Hybrid Seed Production

The main objective of this study was to establish if combining ability of a series of *A. annua* lines, as determined by analysis of a diallel cross, could be used to identify elite parents for the production of improved hybrids. We had previously performed ethyl methane sulphonate mutagenesis on F1 Artemis seed, selfed this material and screened the resulting population to identify individuals with high levels of artemisinin per unit dry weight [12]. Analysis of DNA from this population using the TILLING method [31] revealed a mutation frequency of 1 per 5.4 Mb which is very low compared to the SNP frequency of 1 in 104 bp found in the parental F1 Artemis material [12]. We therefore concluded that the selfed F2 FSHY individuals identified in the forward screen would make suitable parents for hybrid seed production without the need for backcrossing, which is typically required following mutagenesis. In order to maximise the phenotypic diversity within the parental material used in the diallel cross and encourage heterosis, a subset of 30 of the FSHY individuals were selected as parents on the basis of a range of phenotypes displayed when the material was grown under glass (Table S1).

Field evaluation of 130 FSHY lines, including the 30 parental lines subsequently selected for the diallel cross, was carried out in 2008 at Swiss and UK sites and previously reported [12]. Analysis of the data demonstrated that significant genetic variation (between ∼20–50%) existed for all traits measured. This included artemisinin concentration despite the fact that this material had been stringently selected on the basis of artemisinin concentration under glass. This observation was consistent with the fact that phenotypic traits that impact on artemisinin concentration such as trichome density were seen to be variable in material grown under glass and such traits were used to select the subset of parental lines for the diallel cross. The UK and Swiss 2008 field trials of 130 FSHY lines also gave us the opportunity to measure biomass. Significant variation for this trait was important in the selection of parental lines as leaf biomass and leaf to stem ratio are traits that are taken into consideration in generating new hybrids for commercial production of artemisinin [4]. In addition to significant genotype effects, these trials also revealed significant Genotype by Environment interactions for all traits. In general the 130 FSHY lines performed better at the Swiss site than at the UK site, highlighting the potential impact of environmental factors such as temperature, irrigation and altitude on future hybrid performance.

### Evaluation of Hybrid Progeny from Diallel Crosses

It was shown that when grown under glass a number of hybrids outperformed the F1 Artemis variety in terms of both artemisinin concentration and leaf area (Table S4). Furthermore, it was obvious that a small number of the diallel parents such as parents 8 and 30 were overrepresented in the lineage of the high performing hybrids (Figure 2). To further establish the genetic basis of the parental lines that produce high performing hybrids, combining ability analysis was carried out based on Griffing’s method 3 [16]. This method of analysis calculates GCA and SCA from the F1 hybrid progeny and their reciprocals. From this analysis GCA is used to designate the average performance of a parental line and SCA to designate cases in which certain hybrid combinations perform better or worse than would be expected (from the average performance of the parental lines involved). This analysis is similar to that carried out by Delabays on *A. Annua* clones [28]. Our analysis identified significant GCA effects for all traits measured including artemisinin concentration and implies that additive gene action is responsible for the variance in artemisinin related traits observed in the hybrid progeny of the diallel cross when grown under glass. Significant GCA values and high additive gene action were also noted by Delabays [28]. In contrast to the GCA effects, the SCA effects were not significant for any traits measured, implying that non additive gene action or dominance gene effects were not as important as additive effects on hybrid performance. This finding is in contrast to the report of Delabays, where SCA values were significant for artemisinin concentration in *A. annua* clones grown in the field [28].

In contrast with the findings of Delabays [28], reciprocal effects calculated from the analysis of all measured traits in the glasshouse grown hybrid material were found to be significant (Table 2). However, although significant reciprocal effects are seen in 8% of the crosses there are no common parents associated with these. This suggests specific parental effects are an unlikely cause of the differences observed. It is possible that other factors such as missing data values and large error values could erroneously increase the level of significance of reciprocal effects in our calculation particularly since we do not always have data available from both sides of a cross. To further investigate this, data from a 9×9 subset of the parental lines used in the 28 × 28 diallel were re-analysed to reduce the number of missing values in the calculation (Table S9). The combining analysis on this reduced data set showed the error values to remain large and the GCA and reciprocal values still to be significant but there is still no common parent associated with these. Specific localised environmental effects under glasshouse conditions may also account for some of the variation observed in the reciprocal crosses. To assist in the selection of elite parents for production of commercial hybrids from the 28 parental lines used in the diallel cross, BLUP estimates were extracted from the combining ability analysis model to give estimated GCA values for each parent and each trait (Table 3). BLUPs can be used as a method to estimate the random effects from mixed models [32]. Three out of the 28 parental lines analysed were found to have significant positive GCA values for artemisinin concentration. These were parents 8, 10 and 30, with the GCA estimate for parent 30 being the highest. Parents 8 and 30 were also noted to have significant positive GCA values for leaf area (Table 3). Based on the GCA results parents 8 and 30 were identified as lines that would make good parents for future crossing strategies. The GCA values for artemisinin concentration and leaf area in the parental lines correlated well with the performance of hybrid progeny derived from these same parental lines in the 2008 UK and Swiss field trials (Figure 3A and Figure S1A) with parents 8, 10 and 30 appearing consistently as promising parents. Similar correlations were not seen for other traits considered important in the breeding programme highlighting the value of performing the diallel analysis to identify parents with good GCA. Figure 3B and Figure S1B shows how the QTL scores calculated (Table S3) for the parental lines based on the data previously presented [12] also correlate well with the parental GCA values, with parents 8, 10 and 30 again featuring a high number of positive QTLS for artemisinin yield.

### Field Trial Selection

Analysis of hybrid performance was extended from previously reported [12] glasshouse trials in York to experimental field trials at sites in Madagascar and Switzerland in 2009. Trials were performed predominantly on hybrids derived from the diallel cross that had performed well under glass with a significant number being derived from the parents 8, 10 and 30. There was a significant difference between hybrids for all traits recorded at both sites (Table S5). BLUPS extracted from the individual site analysis showed that hybrids derived from parent 30 had the best performance at both sites (Figure 4A&B), consistent with the high GCA value of this line as determined by analysis of data derived from glasshouse grown material. However, the high performance under glass of hybrids derived from parent line 8 which also had high GCA values was not observed in the field. Based on the ranked BLUP values for yield from 2009, out of the 12 hybrids derived from parent 8, only one occurred in the top 10 hybrids in the 2009 Madagascan trial and none ranked in the top 10 in the 2009 Swiss trial (Table S6). Parent 8 did perform well in terms of artemisinin concentration in the 2008 Swiss field trial but the BLUP values for both number of branches and fresh weight were low. This could explain the poor performance of hybrids derived from parent 8 in the experimental field trials, which recorded artemisinin yield (kg/ha). Notably, in the 2009 Madagascar trial which was located in a region used for commercial cultivation of *A. annua*, all but one of the selected hybrids from the diallel cross outperformed the local check (Figure 4B).

The hybrids tested in the 2009 Swiss and Madagascar trials were derived from 21 of the 28 parental lines used in the diallel, with some parental lines represented more frequently than others in the hybrid progeny (Table S7). Despite this, a significant genotype effect was still observed for artemisinin concentration and plant height (Table 4). This suggests that sufficient genetic variation persists amongst this subset of parental lines to allow further gains to be achieved through selective breeding. The G×E effect for this same set of selected hybrids was highly significant not just for artemisinin concentration and plant height but also artemisinin yield and leaf yield at both the Swiss and Madagascar sites (Table 4). The environmental interaction may be due to a combination of differences in climate and temperature and local practices at the two sites, including for example soil conditions, irrigation and post-harvest leaf drying methods. The Madagascar site practices reflected conditions used for commercial production while the Swiss site reflected conditions used for crop breeding. Variation in artemisinin content has previously been attributed to differences in farming practices [5] [10] [4] [11]. *A. annua* is grown in diverse geographic locations ranging from East Africa to Madagascar, India, Vietnam and China. Our results, using a subset of hybrids selected from a diallel cross and field trialled at just two sites in Switzerland and Madagascar, indicate that G×E effects are highly significant over genotype effects alone. This highlights the need to perform field trials at the sites where a hybrid might be grown commercially in order to identify those hybrids that exhibit the best G and G×E interactions.

The approach of selecting parental lines for improved hybrid production based on forward screening and diallel cross analysis of F2 material that carries a relatively high level of natural variation and a low level of induced mutation has been successful. The screening of progeny from our diallel cross under glass has allowed us to identify a selection of parental lines for use in further crosses for hybrid production based on their significant positive GCA values. The screening of the progeny from these parental lines at field trial sites in Switzerland and Madagascar confirmed the performance previously seen under glass. This evidence alongside the GCA values, when combined with QTL information highlighting common parental lines, gave us further confidence in our selection of parental lines for further crosses. The GxE interactions identified from the analysis of parental and hybrid progeny data has also highlighted the importance of trialling hybrids at various locations specific to where selected hybrids would be grown commercially.

## Supporting Information

Figure S1 A) Relationship between parental good combining ability (GCA) values and leaf area measurements recorded from parental lines grown in the field in the UK in 2008. B) Relationship between parental GCA values and QTL scores for parent lines.
(DOCX)

Table S1 Phenotypic characteristics of parental lines selected for inclusion in a 30×30 diallel cross.
(DOCX)

Table S2 Heterozygosity values for the Artemis derived parental lines of the diallel cross. The heterozygosity values for the parents of Artemis C1 and C4 are also included.
(DOCX)

Table S3 The genotype classes for markers associated with QTL for artemisinin yield and leaf area. The trait QTL score assigned to each parental line is calculated by summing the score given for each parent for the marker class – value in the parentheses.
(DOCX)

Table S4 Comparison of leaf area (mm^2^) and artemisinin concentration (µg/mg) recorded from plants grown under glass for hybrid progeny produced from a diallel cross and the F1 hybrid variety, Artemis.
(DOCX)

Table S5 Genotype variation found from the models analysing the Swiss and Madagascan trials independently.
(DOCX)

Table S6 F-values calculated for Site as fixed from the combined model analysing the Swiss and Madagascan trials.
(DOCX)

Table S7 Average performance for hybrids grown at both the Swiss and Madagascan trials. Best linear unbiased predictor (BLUP) values were calculated from a Genotype × Environment analysis.
(DOCX)

Table S8 Average minimum and maximum temperatures and rainfall recorded at the Swiss and Madagascan hybrid field trial sites in 2009.
(DOCX)

Table S9 Combining ability variances calculated for the parental lines of a 9×9 diallel cross grown under glass.
(DOCX)
