# Supplementary material for: The Use of Combining Ability Analysis to Identify Elite Parents for Artemisia annua F1 Hybrid Production
Source: PLoS One. 2013 Apr 23;8(4):e61989. doi: 10.1371/journal.pone.0061989 (PMC3633910; doi:10.1371/journal.pone.0061989)

**Figure S1.** A) Relationship between parental good combining ability (GCA) values and leaf area measurements recorded from parental lines grown in the field in the UK in 2008. B) Relationship between parental GCA values and QTL scores for parent lines.


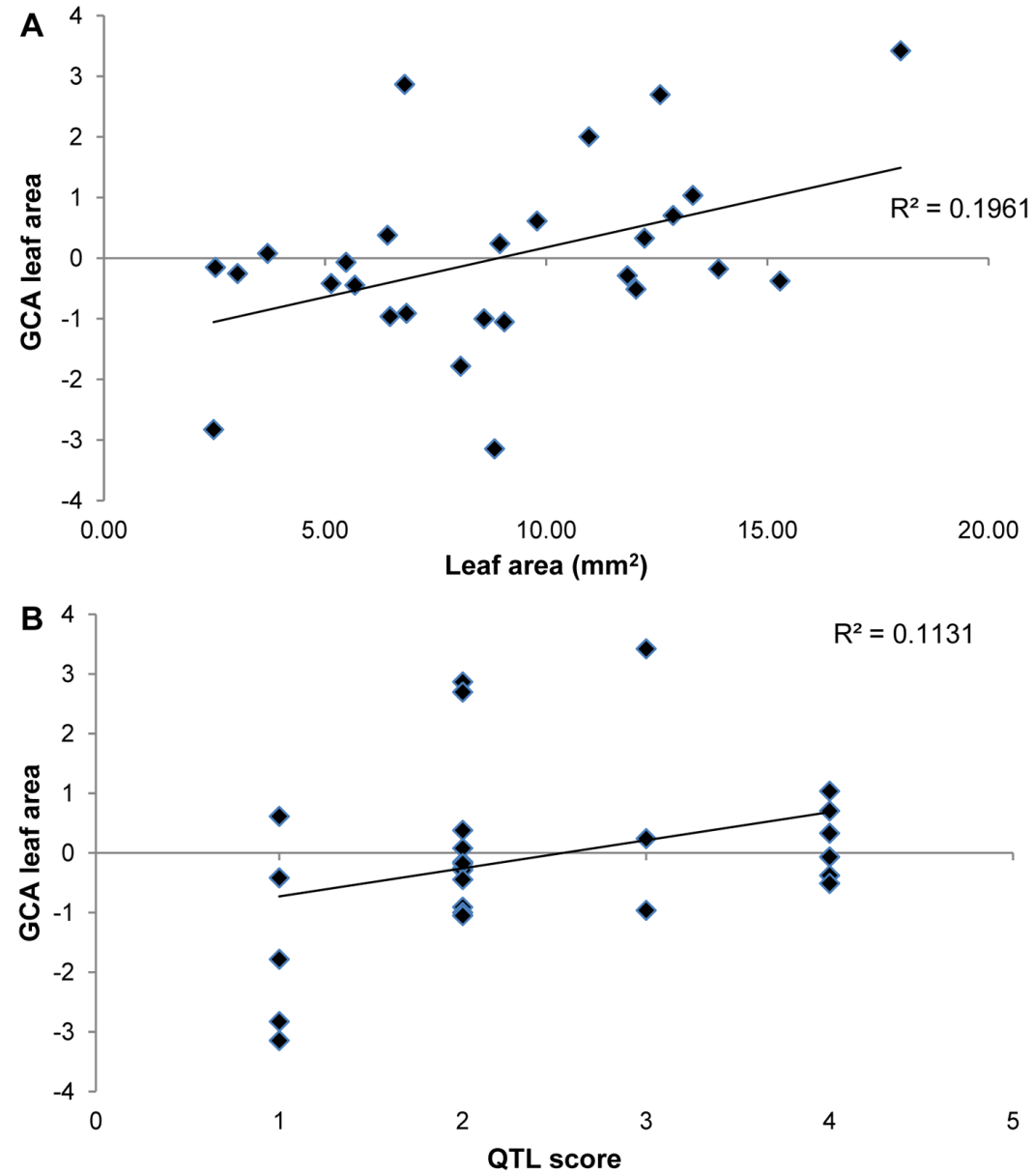

Supplement: Figure S1 — A) Relationship between parental good combining ability (GCA) values and leaf area measurements recorded from parental lines grown in the field in the UK in 2008. B) Relationship between parental GCA values and QTL scores for parent lines. (DOCX) [file pone.0061989.s001.docx]
