# Supplementary material for: The Use of Combining Ability Analysis to Identify Elite Parents for Artemisia annua F1 Hybrid Production
Source: PLoS One. 2013 Apr 23;8(4):e61989. doi: 10.1371/journal.pone.0061989 (PMC3633910; doi:10.1371/journal.pone.0061989)
Supplement: Table S1 — Phenotypic characteristics of parental lines selected for inclusion in a 30×30 diallel cross. (DOCX) [file pone.0061989.s002.docx]

**Table S1.** Phenotypic characteristics of parental lines selected for inclusion in a 30x30 diallel cross.

| **Parent** | **Flowering** | **Leaf area** | **Trichome density** |
| --- | --- | --- | --- |
| 1 | average | large | average |
| 2 | average | average | average |
| 3 | early | average | low |
| 4 | early | average | average |
| 5 | average | small | average |
| 6 | late | small | high |
| 7 | average | large | average |
| 8 | average | average | high |
| 9 | late | small | high |
| 10 | average | average | average |
| 11 | late | large | average |
| 12 | late | average | average |
| 13 | late | small | low |
| 14 | late | average |  |
| 15 | average | large | average |
| 16 | late | large | average |
| 17 |  | average |  |
| 18 | late | average | high |
| 19 | late | average | average |
| 20 | early | average | average |
| 21 | average | average | high |
| 22 |  | small | average |
| 23 |  | small | average |
| 24 | average | average | low |
| 25 |  | average | high |
| 26 | average |  |  |
| 27 |  | average |  |
| 28 | early | large | average |
| 29 | early | average | high |
| 30 | average | large | high |
|  |  |  |  |

Mean and quartile values were calculated for thirty parental lines following cultivation under glass for 12.5 weeks. Results were considered to be *average* if they fell between the first and third quartiles, *high* (or *late*, in the case of flowering) if they were above the third quartile and *low* (or *early*, in the case of flowering) if they were below the first quartile.
