# Supplementary material for: The Use of Combining Ability Analysis to Identify Elite Parents for Artemisia annua F1 Hybrid Production
Source: PLoS One. 2013 Apr 23;8(4):e61989. doi: 10.1371/journal.pone.0061989 (PMC3633910; doi:10.1371/journal.pone.0061989)
Supplement: Table S2 — Heterozygosity values for the Artemis derived parental lines of the diallel cross. The heterozygosity values for the parents of Artemis C1 and C4 are also included. (DOCX) [file pone.0061989.s003.docx]

**Table S2.** Heterozygosity values for the Artemis derived parental lines of the diallel cross. The heterozygosity values for the parents of Artemis C1 and C4 are also included.

| Parent_id | Hz values |
| --- | --- |
| C1 | 0.43 |
| C4 | 0.65 |
| 1 | 0.43 |
| 2 | 0.44 |
| 3 | 0.42 |
| 4 | 0.31 |
| 5 | 0.32 |
| 6 | 0.35 |
| 7 | 0.16 |
| 8 | 0.29 |
| 9 | 0.27 |
| 10 | 0.31 |
| 11 | 0.45 |
| 12 | 0.48 |
| 13 | 0.30 |
| 14 | 0.34 |
| 15 | 0.29 |
| 16 | 0.32 |
| 17 | 0.40 |
| 18 | 0.36 |
| 19 | 0.48 |
| 20 | 0.33 |
| 21 | 0.41 |
| 22 | 0.34 |
| 24 | 0.16 |
| 25 | 0.30 |
| 26 | 0.22 |
| 27 | 0.31 |
| 28 | 0.23 |
| 29 | 0.45 |
| 30 | 0.26 |
