# Supplementary material for: The Use of Combining Ability Analysis to Identify Elite Parents for Artemisia annua F1 Hybrid Production
Source: PLoS One. 2013 Apr 23;8(4):e61989. doi: 10.1371/journal.pone.0061989 (PMC3633910; doi:10.1371/journal.pone.0061989)
Supplement: Table S3 — The genotype classes for markers associated with QTL for artemisinin yield and leaf area. The trait QTL score assigned to each parental line is calculated by summing the score given for each parent for the marker class – value in the parentheses. (DOCX) [file pone.0061989.s004.docx]

**Table S3.** The genotype classes for markers associated with QTL for artemisinin yield and leaf area. The trait QTL score assigned to each parental line is calculated by summing the score given for each parent for the marker class – value in the parentheses.

|  | **Markers associated to QTL for artemisnin yield** | | | | | **Markers associated to QTL for leaf area** | | | | | |
| --- | --- | --- | --- | --- | --- | --- | --- | --- | --- | --- | --- |
| **Parent** | **A32488_367** | **A25506_480** | **A44606_551** | **B2032_381** | **Yield QTL score** | **A28035_469** | **A5495_225** | **A2822_572** | **A9883_401** | **U6536_2512** | **Leaf area QTL score** |
| **C1** | BB(0) | AB(1) | AB(1) | AA(0) | 2 | BB(0) | AB(1) | BB(0) | AA(0) | BB(0) | 1 |
| **C4** | AB(1) | AA(0) | AA(0) | AB(1) | 2 | AB(1) | BB(0) | AB(1) | AB(1) | AB(1) | 4 |
| **1** | AB(1) | AA(0) | AB(1) | AB(1) | 3 | BB(0) | AB(1) | AB(1) | AB(1) | AB(1) | 4 |
| **2** | BB(0) | AA(0) | AB(1) | AB(1) | 2 | AA(1) | AB(1) | AA(1) | AB(1) | BB(0) | 4 |
| **3** | AB(1) | AB(1) | AB(1) | AA(0) | 3 | AB(1) | BB(0) | AB(1) | AA(0) | BB(0) | 2 |
| **4** | BB(0) | BB(1) | AA(0) | AA(0) | 1 | AB(1) | BB(0) | BB(0) | AA(0) | BB(0) | 1 |
| **5** | BB(0) | AA(0) | AB(1) | AA(0) | 1 | BB(0) | BB(0) | AB(1) | AA(0) | BB(0) | 1 |
| **6** | AB(1) | AA(0) | AA(0) | BB(1) | 2 | BB(0) | BB(0) | AB(1) | BB(1) | BB(0) | 2 |
| **7** | AA(1) | AA(0) | BB(1) | BB(1) | 3 | AA(1) | BB(0) | AA(1) | AB(1) | AB(1) | 4 |
| **8** | BB(0) | AB(1) | AB(1) | AB(1) | 3 | BB(0) | BB(0) | AB(1) | AA(0) | AB(1) | 2 |
| **9** | AB(1) | AA(0) | AB(1) |  | 2 | BB(0) | AB(1) | BB(0) | AB(1) | BB(0) | 2 |
| **10** | AA(1) | AA(0) | AB(1) | AA(0) | 2 | BB(0) | BB(0) | AA(1) | BB(1) | BB(0) | 2 |
| **11** | BB(0) | AB(1) | AB(1) | AB(1) | 3 | AA(1) | BB(0) | AB(1) | AB(1) | BB(0) | 3 |
| **12** | AA(1) | AA(0) | AB(1) | AA(0) | 2 | BB(0) | AB(1) | BB(0) | AA(0) | BB(0) | 1 |
| **13** | BB(0) | AA(0) | AB(1) | AB(1) | 2 | BB(0) | BB(0) | AB(1) | AA(0) | AB(1) | 2 |
| **14** | AB(1) | AB(1) | AA(0) | AB(1) | 3 | AB(1) | BB(0) | AB(1) | AA(0) | BB(0) | 2 |
| **15** | AB(1) | AA(0) | AA(0) | AA(0) | 1 | AB(1) | AB(1) | AA(1) | AB(1) | BB(0) | 4 |
| **16** | AA(1) | AB(1) | AB(1) | AA(0) | 3 | BB(0) | BB(0) | BB(0) | AB(1) | AB(1) | 2 |
| **17** | AB(1) | AB(1) | AB(1) | AB(1) | 4 | AB(1) | AB(1) | BB(0) | AB(1) | AA(1) | 4 |
| **18** | BB(0) | AB(1) | AA(0) | AB(1) | 2 | AA(1) | BB(0) | BB(0) | AB(1) | AB(1) | 3 |
| **19** | BB(0) | AA(0) | AA(0) | AB(1) | 1 | BB(0) | BB(0) | BB(0) | AB(1) | BB(0) | 1 |
| **20** | BB(0) | AB(1) | BB(1) | AA(0) | 2 | BB(0) | BB(0) | BB(0) | AB(1) | BB(0) | 1 |
| **21** | AB(1) | AA(0) | AA(0) | AA(0) | 1 | BB(0) | BB(0) | AB(1) | AA(0) | AB(1) | 2 |
| **22** | AA(1) | AA(0) | AB(1) | AA(0) | 2 | AB(1) | BB(0) | AB(1) | AA(0) | BB(0) | 2 |
| **24** | BB(0) | AA(0) | BB(1) | AA(0) | 1 | BB(0) | BB(0) | AA(1) | BB(1) | BB(0) | 2 |
| **25** | AA(1) | AA(0) | AB(1) | AB(1) | 3 | BB(0) | BB(0) | AB(1) | BB(1) | AB(1) | 3 |
| **26** | BB(0) | AA(0) | AA(0) | AB(1) | 1 | BB(0) | AB(1) | AA(1) | AA(0) | BB(0) | 2 |
| **27** | AA(1) | AA(0) | AA(0) | AA(0) | 1 | AB(1) | BB(0) | AA(1) | AB(1) | BB(0) | 3 |
| **28** | BB(0) | AB(1) | BB(1) | AB(1) | 3 | AA(1) | AB(1) | AB(1) | AA(0) | AA(1) | 4 |
| **29** | AB(1) | AB(1) | AA(0) | AA(0) | 2 | BB(0) | BB(0) | BB(0) | AA(0) | BB(0) | 0 |
| **30** | AA(1) | BB(1) | AB(1) | AA(0) | 3 | BB(0) | BB(0) | AA(1) | AB(1) | BB(0) | 2 |
