# Supplementary material for: The Use of Combining Ability Analysis to Identify Elite Parents for Artemisia annua F1 Hybrid Production
Source: PLoS One. 2013 Apr 23;8(4):e61989. doi: 10.1371/journal.pone.0061989 (PMC3633910; doi:10.1371/journal.pone.0061989)
Supplement: Table S4 — Comparison of leaf area (mm2) and artemisinin concentration (µg/mg) recorded from plants grown under glass for hybrid progeny produced from a diallel cross and the F1 hybrid variety, Artemis. (DOCX) [file pone.0061989.s005.docx]

**Table S4.** Comparison of leaf area (mm^2^) and artemisinin concentration (µg/mg) recorded from plants grown under glass for hybrid progeny produced from a diallel cross and the F1 hybrid variety, Artemis.

| **Plants** | **Trait** | **Minimum** | **Maximum** | **Average** | **Standard deviation** |
| --- | --- | --- | --- | --- | --- |
| Hybrids | Artemisinin concentration | 0.82 | 37.42 | 10.62 | 4.13 |
| Artemis | Artemisinin concentration | 2.74 | 29.79 | 10.48 | 3.10 |
| Hybrids | Leaf area | 117.92 | 3388.62 | 1309.55 | 534.45 |
| Artemis | Leaf area | 566.467 | 2413.33 | 1201.60 | 410.91 |
