# Supplementary material for: The Use of Combining Ability Analysis to Identify Elite Parents for Artemisia annua F1 Hybrid Production
Source: PLoS One. 2013 Apr 23;8(4):e61989. doi: 10.1371/journal.pone.0061989 (PMC3633910; doi:10.1371/journal.pone.0061989)
Supplement: Table S5 — Genotype variation found from the models analysing the Swiss and Madagascan trials independently. (DOCX) [file pone.0061989.s006.docx]

**Table S5.** Genotype variation found from the models analysing the Swiss and Madagascan trials independently.

|  |  | **Plant leaf dry weight (g)** | **Leaf yield kg/ha** | **Artemisinin concentration (µg/mg)** | **Yield kg/ha** | **Average height (cm)** |
| --- | --- | --- | --- | --- | --- | --- |
| **Switzerland** | Genotype | 180.17** | 48269.00* | 0.63*** | 17.22*** | 173.38*** |
|  | Residual | 370.81 | 131345.00 | 0.75 | 15.42 | 161.41 |
| **Madagascar** | Genotype | 81.19** | 19217.00** | 1.12*** | 4.73*** | 119.99**** |
|  | Residual | 229.07 | 54218.00 | 0.67 | 7.95 | 54.10 |

* indicates significance at 0.05 level, ** indicates significance at 0.01 level, *** indicates significance at 0.001 and **** indicates significance at <0.001
