# Supplementary material for: The Use of Combining Ability Analysis to Identify Elite Parents for Artemisia annua F1 Hybrid Production
Source: PLoS One. 2013 Apr 23;8(4):e61989. doi: 10.1371/journal.pone.0061989 (PMC3633910; doi:10.1371/journal.pone.0061989)
Supplement: Table S6 — F-values calculated for Site as fixed from the combined model analysing the Swiss and Madagascan trials. (DOCX) [file pone.0061989.s007.docx]

**Table S6.** F-values calculated for Site as fixed from the combined model analysing the Swiss and Madagascan trials.

|  | **Average plant leaf dry weight (g)** | **Average leaf yield kg/ha** | **Average artemisinin concentration (µg/mg)** | **Average yield kg/ha** | **Average height (cm)** |
| --- | --- | --- | --- | --- | --- |
| **Switzerland** | 160.58 (23.80) | 2867.55 (425.09) | 1.11 (0.12) | 31.84 (5.85) | 167.55 (21.78) |
| **Madagascar** | 95.11 (18.98) | 1463.06 (291.95) | 1.02 (0.14) | 15.11 (4.03) | 129.41 (14.67) |
| **F-test value** | 207.31**** | 331.24**** | 16.85*** | 473.49**** | 169.39**** |

Values in parentheses are standard deviation, *** indicates significance at 0.001 level and **** indicates significance at <0.001 level
