# Supplementary material for: The Use of Combining Ability Analysis to Identify Elite Parents for Artemisia annua F1 Hybrid Production
Source: PLoS One. 2013 Apr 23;8(4):e61989. doi: 10.1371/journal.pone.0061989 (PMC3633910; doi:10.1371/journal.pone.0061989)
Supplement: Table S7 — Average performance for hybrids grown at both the Swiss and Madagascan trials. Best linear unbiased predictor (BLUP) values were calculated from a Genotype × Environment analysis. (DOCX) [file pone.0061989.s008.docx]

**Table S7.** Average performance for hybrids grown at both the Swiss and Madagascan trials. Best linear unbiased predictor (BLUP) values were calculated from a Genotype x Environment analysis.

|  | | | **Switzerland** | | | | | | **Madagascar** | | | | | |
| --- | --- | --- | --- | --- | --- | --- | --- | --- | --- | --- | --- | --- | --- | --- |
| Hybrid ID | Mother parent line | Father parent line | Average leaf yield kg/ha | BLUP leaf yield kg/ha | Average artemisinin concentration (µg/mg) | BLUP artemisinin concentration (µg/mg) | Average yield kg/ha | BLUP yield kg/ha | Average leaf yield kg/ha | BLUP leaf yield kg/ha | Average artemisinin concentration (µg/mg) | BLUP artemisinin concentration (µg/mg) | Average yield kg/ha | BLUP yield kg/ha |
| Artemis |  |  | 3123.21 | 86.33 | 1.05 | 0.27 | 32.70 | 2.14 | 1562.31 | 38.51 | 0.89 | -0.86 | 13.94 | -0.88 |
| hyb1003r | 30 | 3 | 2979.46 | 88.42 | 1.18 | -0.06 | 34.91 | 2.62 | 1250.26 | -117.95 | 0.91 | -1.73*** | 11.75 | -2.79 |
| hyb1005 | 11 | 10 | 2918.75 | 4.80 | 1.13 | 0.73 | 33.11 | -0.01 | 1572.31 | -5.92 | 1.08 | -0.86 | 17.02 | 0.35 |
| hyb1005r | 10 | 11 | 3348.21 | 143.90 | 1.22 | 0.00 | 40.40 | 6.50*** | 1192.31 | -131.47 | 0.92 | 0.11 | 10.91 | -3.05 |
| hyb1006 | 30 | 13 | 2525.89 | -92.88 | 1.18 | 0.90 | 30.00 | -1.08 | 1452.82 | 1.15 | 1.23 | -0.77 | 17.85 | 1.17 |
| hyb1015 | 29 | 11 | 2973.21 | 61.54 | 1.01 | 0.17 | 29.83 | -1.42 | 1537.95 | 45.19 | 0.96 | 0.79 | 14.74 | 0.99 |
| hyb1015r | 11 | 29 | 2734.82 | 55.39 | 1.17 | -0.56 | 31.98 | 1.94 | 1606.67 | 40.03 | 1.06 | -0.03 | 16.95 | 0.78 |
| hyb1016r | 24 | 11 | 2974.11 | 5.75 | 1.11 | 0.34 | 32.87 | 0.58 | 1417.95 | -17.29 | 0.83 | 0.05 | 12.06 | -2.08 |
| hyb1017 | 11 | 3 | 2801.79 | -30.41 | 1.24 | 0.30 | 34.78 | 1.45 | 1551.28 | 20.94 | 0.86 | -1.07* | 13.51 | -2.18 |
| hyb1023 | 4 | 30 | 3157.14 | 85.21 | 1.05 | 1.03 | 33.07 | 0.89 | 1563.59 | 11.39 | 1.09 | -1.33** | 17.06 | 0.97 |
| hyb1052 | 4 | 8 | 3008.04 | 50.25 | 0.94 | -0.33 | 28.28 | -1.18 | 1739.49 | 55.29 | 0.93 | 0.37 | 16.21 | 0.46 |
| hyb1052r | 8 | 4 | 3269.64 | 63.28 | 0.96 | -0.42 | 31.37 | -0.46 | 1929.23 | 151.95 | 0.95 | -0.41 | 18.11 | 1.95 |
| hyb1053 | 7 | 11 | 2779.46 | -105.84 | 1.19 | -0.43 | 33.20 | 0.01 | 1449.23 | 30.60 | 1.02 | -0.31 | 14.99 | -0.50 |
| hyb1053r | 11 | 7 | 2605.36 | -34.70 | 1.27 | 0.38 | 32.86 | -0.02 | 1337.44 | -11.54 | 0.98 | -0.25 | 13.21 | -0.61 |
| hyb1062 | 8 | 11 | 3041.96 | 29.51 | 0.98 | 0.69 | 29.82 | -1.26 | 1184.10 | -81.36 | 1.01 | -0.40 | 12.02 | -1.57 |
| hyb1064 | 5 | 30 | 3166.07 | 77.78 | 1.10 | -0.57 | 34.84 | 1.89 | 1350.77 | -28.71 | 0.90 | 0.17 | 12.38 | -1.42 |
| hyb1074 | 30 | 14 | 3264.29 | 95.99 | 1.20 | 0.02 | 39.05 | 3.25 | 1454.36 | -32.13 | 1.13 | -0.53 | 16.48 | 0.51 |
| hyb1077 | 30 | 29 | 3059.82 | 16.57 | 1.13 | 0.27 | 34.37 | 1.13 | 1710.77 | 43.90 | 1.10 | 0.43 | 18.89 | 1.75 |
| hyb1077r | 29 | 30 | 3169.64 | 99.90 | 1.06 | -0.02 | 33.76 | 1.43 | 1388.21 | -19.30 | 1.06 | 0.32 | 14.66 | -0.19 |
| hyb1078r | 20 | 8 | 3191.96 | 86.65 | 0.94 | -0.44 | 30.15 | -0.35 | 1581.03 | 30.39 | 0.95 | 0.40 | 15.29 | -0.11 |
| hyb1079 | 30 | 7 | 2733.93 | -44.58 | 1.13 | -0.54 | 30.89 | 0.07 | 1342.05 | -0.98 | 1.11 | -0.31 | 14.72 | 0.46 |
| hyb1079r | 7 | 30 | 2782.14 | 16.40 | 1.17 | -0.06 | 32.62 | 0.95 | 1243.59 | -62.96 | 1.13 | 0.58 | 14.04 | -0.09 |
| hyb1082 | 8 | 2 | 2056.25 | -189.69 | 0.95 | 0.07 | 19.43 | -7.24*** | 740.00 | -176.79 | 0.80 | 0.60 | 5.92 | -4.77*** |
| hyb1082r | 2 | 8 | 2616.07 | -67.24 | 1.07 | -0.41 | 28.26 | -1.34 | 1487.69 | -13.54 | 1.00 | -0.87 | 14.99 | -0.39 |
| hyb1083r | 10 | 8 | 2624.11 | -90.57 | 1.08 | 0.09 | 28.72 | -2.01 | 1427.18 | -19.98 | 1.03 | -0.21 | 14.78 | -0.64 |
| hyb1085 | 2 | 30 | 2832.14 | 14.49 | 1.07 | -0.02 | 30.14 | -1.14 | 1787.69 | 71.00 | 1.17 | -0.07 | 20.73 | 2.55 |
| hyb1086 | 19 | 30 | 3501.79 | 132.79 | 1.21 | -0.56 | 42.15 | 6.61*** | 1501.03 | -19.68 | 1.05 | 0.68 | 15.81 | -0.08 |
| hyb1101 | 12 | 30 | 1982.14 | -248.60 | 1.12 | 0.48 | 22.48 | -5.43* | 1829.23 | 160.94 | 1.10 | 0.06 | 20.16 | 3.74* |
| hyb1101r | 30 | 12 | 2508.93 | -47.01 | 1.13 | 0.02 | 28.26 | -0.33 | 1643.59 | 74.96 | 1.12 | 0.36 | 18.60 | 2.73 |
| hyb1102 | 30 | 10 | 2866.96 | -9.04 | 1.16 | 0.05 | 33.22 | 0.16 | 1691.79 | 64.48 | 1.17 | 0.53 | 19.73 | 2.52 |
| hyb1107 | 30 | 8 | 2588.39 | -14.70 | 1.22 | 0.12 | 31.27 | 0.11 | 1166.67 | -69.49 | 1.08 | 0.61 | 12.61 | -0.96 |
| hyb1113 | 30 | 11 | 2937.50 | -42.49 | 1.15 | 0.18 | 33.61 | -0.19 | 1380.00 | 5.84 | 1.17 | 0.26 | 16.21 | 1.63 |
| hyb1113r | 11 | 30 | 2813.39 | -20.19 | 1.20 | -0.30 | 33.64 | 0.90 | 1508.21 | 36.56 | 1.10 | 1.00 | 16.52 | 1.42 |
| hyb1116r | 28 | 30 | 2825.89 | -46.29 | 1.28 | 0.41 | 35.65 | 1.34 | 1452.82 | -6.56 | 1.22 | 0.26 | 17.80 | 1.11 |
| hyb1117 | 26 | 8 | 2236.61 | -170.98 | 0.91 | 0.47 | 20.36 | -6.72*** | 1183.59 | -28.40 | 1.01 | 0.79 | 11.93 | -0.86 |
| hyb1130 | 8 | 1 | 2692.86 | -21.90 | 1.01 | -1.05 | 27.09 | -3.06 | 1406.15 | -47.00 | 0.97 | 0.36 | 13.81 | -0.90 |
| hyb1130r | 1 | 8 | 2712.50 | -37.31 | 0.99 | -0.49 | 26.98 | -3.02 | 1217.95 | -67.16 | 0.96 | -0.17 | 11.76 | -1.64 |
| hyb1134 | 30 | 21 | 2795.54 | 20.16 | 1.30 | -0.47 | 36.27 | 3.93 | 1551.79 | 7.16 | 1.13 | -0.10 | 17.49 | 0.21 |
| hyb1137 | 1 | 11 | 2885.71 | -14.73 | 1.12 | 0.82 | 32.25 | -0.23 | 1282.05 | -57.17 | 0.89 | 0.08 | 11.45 | -2.33 |
| hyb1137r | 11 | 1 | 2821.43 | -29.22 | 1.19 | 0.17 | 33.65 | 0.90 | 1234.87 | -73.03 | 0.99 | -0.66 | 12.17 | -1.95 |
| hyb1141 | 29 | 8 | 2512.50 | -133.60 | 1.02 | 0.48 | 25.46 | -3.80 | 1533.85 | 30.77 | 1.02 | -0.33 | 15.72 | 0.30 |
| hyb1141r | 8 | 29 | 2700.00 | -99.72 | 0.99 | -0.59 | 26.70 | -4.09* | 1721.03 | 101.30 | 1.05 | 0.05 | 18.04 | 1.50 |
| hyb1153 | 26 | 30 | 2143.75 | -155.10 | 1.09 | -0.81 | 23.27 | -4.49* | 1670.77 | 54.14 | 1.17 | 0.23 | 19.63 | 2.48 |
| hyb1153r | 30 | 26 | 3098.21 | 37.71 | 1.03 | -0.26 | 31.67 | -0.08 | 1666.15 | 78.88 | 1.12 | 0.61 | 18.65 | 2.74 |
| hyb1174 | 1 | 30 | 3016.07 | 37.90 | 1.14 | -0.37 | 34.30 | 1.38 | 1612.31 | 15.08 | 1.19 | 0.65 | 19.28 | 1.40 |
| hyb1174r | 30 | 1 | 3243.75 | 166.64 | 1.31 | -0.21 | 42.53 | 5.64** | 1406.15 | -60.89 | 1.13 | 0.64 | 15.92 | -0.55 |
| hyb1187 | 27 | 30 | 2945.54 | 71.10 | 1.05 | 0.76 | 31.10 | -0.46 | 1384.62 | -19.48 | 1.00 | 0.18 | 13.84 | 0.05 |
| Local check |  |  | 3415.63 | 198.35 | 1.07 | -0.28 | 36.80 | 3.60* | 1435.90 | -1.71 | 0.64 | 0.09 | 9.27 | -3.26 |

* indicates significance at 0.05 level, ** indicates significance at 0.01 level, *** indicates significance at 0.001 and **** indicates significance at <0.001respectively
