# Supplementary material for: The Use of Combining Ability Analysis to Identify Elite Parents for Artemisia annua F1 Hybrid Production
Source: PLoS One. 2013 Apr 23;8(4):e61989. doi: 10.1371/journal.pone.0061989 (PMC3633910; doi:10.1371/journal.pone.0061989)
Supplement: Table S8 — Average minimum and maximum temperatures and rainfall recorded at the Swiss and Madagascan hybrid field trial sites in 2009. (DOCX) [file pone.0061989.s009.docx]

**Table S8.** Average minimum and maximum temperatures and rainfall recorded at the Swiss and Madagascan hybrid field trial sites in 2009.

|  | **Switzerland** | | | **Madagascar** | | |
| --- | --- | --- | --- | --- | --- | --- |
|  | Mean min temp (°C) | Mean max temp (°C) | Mean rainfall (mm) | Mean min temp (°C) | Mean max temp (°C) | Mean rainfall (mm) |
| April | 5.64 | 18.92 | 1.63 |  |  |  |
| May | 9.74 | 23.55 | 0.87 | 9.67 | 25.67 | 0.53 |
| June | 11.44 | 24.91 | 1.30 | 6.80 | 24.57 | 0.87 |
| July | 14.11 | 26.75 | 3.48 | 4.74 | 20.69 | 1.40 |
| August | 14.36 | 28.21 | 0.74 | 8.97 | 25.65 | 1.16 |
| September | 10.34 | 23.66 | 0.63 | 9.10 | 26.13 | 0.50 |
| October | 4.11 | 16.93 | 0.48 | 11.19 | 27.10 | 3.77 |
| November |  |  |  | 13.10 | 27.57 | 3.70 |
