# Supplementary material for: The Use of Combining Ability Analysis to Identify Elite Parents for Artemisia annua F1 Hybrid Production
Source: PLoS One. 2013 Apr 23;8(4):e61989. doi: 10.1371/journal.pone.0061989 (PMC3633910; doi:10.1371/journal.pone.0061989)
Supplement: Table S9 — Combining ability variances calculated for the parental lines of a 9×9 diallel cross grown under glass. (DOCX) [file pone.0061989.s010.docx]

**Table S9.** Combining ability variances calculated for the parental lines of a 9x9 diallel cross grown under glass.

| **Source** | **Artemisinin concentration (µg/mg)** | **Height (cm)** | **Branch number** | **Internode number** | **Leaf area (mm^2^)** | **Trichome density (cm^2^)** |
| --- | --- | --- | --- | --- | --- | --- |
| GCA | 1.61* | 22.91 | 1.77 | 0.02* | 63429.00* | 1.25 |
| SCA | 0.05 | 0.00 | 0.00 | 0.00 | 9811.59 | 0.73 |
| Reciprocal | 2.27** | 65.16**** | 5.47**** | 0.04**** | 30297.00* | 1.85 |
| Error | 14.46 | 220.51 | 24.71 | 0.13 | 144285.00 | 22.70 |

* indicates significance at 0.05 level, ** indicates significance at 0.01 level, *** indicates significance at 0.001 and **** indicates significance at <0.001respectively
